# Cost analysis of IPv6 distributed mobility management protocols in comparison with TFMIPv6

**DOI:** 10.1371/journal.pone.0306132

**Published:** 2024-08-07

**Authors:** Madeeha Aman, Muhammad Zubair, Afzal Badshah, Haleem Farman, Ghani Ur Rehman, Sohaib Bin Altaf Khattak, Moustafa M. Nasralla

**Affiliations:** 1 Department of Computer Science, University of the Punjab Lahore, Lahore, Pakistan; 2 Department of Computer Science and Bioinformatics, Khushal Khan Khattak University, Karak, Pakistan; 3 Department of Computer Science, Hamdard University, Islamabad Campus, Islamabad, Pakistan; 4 Smart Systems Engineering Lab, College of Engineering, Prince Sultan University, Riyadh, Saudi Arabia; Abdul Wali Khan University Mardan, PAKISTAN

## Abstract

The past decade has witnessed a significant evolution in the role of the Internet, transitioning from individual connectivity to an integral aspect of various domains. This shift has prompted a move in IP paradigms from hierarchical to distributed architectures characterized by decentralized structures. This transition empowers efficient data routing and management across diverse networks. However, traditional distributed mobility management (DMM) protocols, reliant on tunneling mechanisms, incur overheads, costs, and delays, exacerbating challenges in managing the exponential growth of mobile data traffic. This research proposes Tunnel-Free Mobility for IPv6 (TFMIPv6) as a solution to address the shortcomings of existing DMM protocols. TFMIPv6 eliminates the need for tunneling, simplifying routing processes and reducing latency. A comprehensive cost analysis and performance evaluation are conducted, comparing TFMIPv6 with traditional protocols such as MIPv6, PMIPv6, FMIPv6, and HMIPv6. The study reveals significant improvements with TFMIPv6. Signaling costs are reduced by 50%, packet delivery costs by 23%, and tunneling costs are completely eliminated (100%). Real-world network traffic datasets are used for simulation, providing statistical evidence of TFMIPv6’s efficacy in supporting an uninterrupted movement of IPv6 data across networks.

## Introduction

In the past decade, we have seen a remarkable transformation in the Internet’s role from being a tool for individual connectivity to becoming an integral part of everything around us. This evolution has spurred a shift in the IP paradigm from traditional hierarchical and centralized network architectures towards more flat and distributed structures [1]. As mobile data traffic grows exponentially, mobile operators face the daunting task of handling this surge. To mitigate these challenges, operators are increasingly turning to data offloading technologies within 3GPP networks, utilizing mechanisms such as Selected IP Traffic Offload (SIPTO) and Local IP Access (LIPA) [2–4].

Despite these advancements, the rapid increase in mobile traffic threatens to outpace the capabilities of current centralized mobility management (CMM) systems, which are plagued by scalability issues such as sub-optimal routing, single points of failure and inefficient use of mobility resources [5, 6]. Distributed mobility management (DMM) has been proposed as a solution, endorsing a flatter network architecture with distributed entities to address these issues. DMM potentially resolves problems like sub-optimal routing and single-point failures [7, 8]. However, despite its benefits in reducing handover delays, DMM faces challenges such as excessive control signalling and tunnelling overheads [9, 10].

Several studies have investigated DMM solutions, focusing on the efficient management of mobile video traffic and other performance improvements [5, 11]. However, a lack of comprehensive overviews fully addresses the key aspects required for effective DMM solution development. Existing literature often offers limited comparisons and narrow assessments [12, 13]. Various studies have conducted cost analyses of mobility protocols under different scenarios, but these, too, are often narrowly focused and do not provide a holistic view of mobility patterns, protocols, and network topologies [14–23]. The authors in [24] provide an in-depth review of the challenges and potential solutions for mobility management in 5G and future network technologies. It discusses the complexities of maintaining seamless connectivity and efficient network resource allocation as mobile devices move across different network segments and evaluate various strategies or advancements to address these challenges. This study [25] presents a well-structured framework to assess the merits and limitations of distributed and centralized mobility management protocols. The focus lies on their efficiency, scalability, and impact on overall network performance. Through a balanced analysis, the paper contrasts the strengths and weaknesses of each approach, providing valuable insights into their suitability across diverse networking scenarios, especially against the backdrop of rapidly advancing mobile technologies. Meanwhile, the study in [26, 27] delves into the readiness of current mobility management solutions in the face of the advanced demands of 5G and beyond technologies. It evaluates existing protocols and infrastructures, scrutinizes their capacity to handle the high-speed, low-latency, and high-density requirements of next-generation networks, and suggests areas for improvement or further development to ensure these systems can support the future of mobile connectivity effectively.

In response to these gaps, our research introduces a framework for a tunnel-free protocol supporting DMM in mobile networks. This framework is designed to enhance communication and minimize delays by eliminating the need for tunnelling, thereby reducing registration delays. Our approach demonstrates significant improvements, including reducing handover delay, blocking probability, and data packet loss [28]. This paper will analyze and compare IP mobility management protocols developed by the IETF with our proposed TFMIPv6 (tunnel-free mobile IPv6) protocol, focusing on a comprehensive cost analysis. This will provide insights into the strengths and weaknesses of each system, offering a more nuanced understanding of their respective advantages and limitations.

### Novelty of TFMIPv6

1. **Tunnel-Free Approach:** TFMIPv6 eliminates the need for tunneling, reducing registration delays and operational costs. This feature provides a significant advantage over existing distributed mobility management protocols like MIPv6, FMIPv6, and PMIPv6, which rely on tunneling.
2. **Cost-Efficiency:** Our research demonstrates that TFMIPv6 achieves reductions of 50% in signaling costs, 23% in packet delivery costs, 100% in tunneling costs, and 13% in total costs compared to traditional protocols.
3. **Make-Before-Break (MBB) Methodology:** TFMIPv6 employs an MBB methodology to minimize packet loss and ensure uninterrupted connectivity during handovers.

### Differences from existing schemes

1. **MIPv6 and FMIPv6:** Rely on Binding Update (BU) messages to inform the Home Agent and Correspondent Node of changes, incurring high signaling and tunneling costs. TFMIPv6 avoids these costs with the Binding Mobility Anchor (BMA).
2. **HMIPv6 and PMIPv6:** Use local management to reduce signaling, but tunneling costs remain significant. TFMIPv6 manages mobility within a tunnel-free domain, providing a cost-effective solution.

The Table 1 compares TFMIPv6 with existing protocols across key performance metrics.

**Table 1:** Comparison of TFMIPv6 with existing protocols.

Protocol	Signaling Cost	Packet Delivery Cost	Tunneling Cost	Total Cost
**TFMIPv6**	Lower	Lower	None	Lowest
**MIPv6**	Higher	Moderate	Higher	Higher
**FMIPv6**	High	High	High	Highest
**HMIPv6**	Moderate	Moderate	Moderate	Moderate
**PMIPv6**	Moderate	Moderate	Moderate	Moderate

Our analysis provides strong evidence that TFMIPv6’s tunnel-free approach is a novel solution for distributed mobility management. The significant reductions in signaling, packet delivery, and total costs, combined with uninterrupted connectivity, position TFMIPv6 as a superior alternative. The following are the main contributions of this article:

- The distributed nature of the protocol could lead to a reduction in signalling overheads, contributing to overall cost efficiency.
- Streamlining data movement to reduce operational expenses is a key consideration for network providers.
- The protocol presents a model for evaluating long-term cost benefits, emphasizing sustained operational savings over time.
- The approach advocates for more efficient use of network resources, thereby reducing unnecessary expenditures.
- The protocol’s cost-effectiveness is highlighted through comparative analyses with traditional network models, illustrating its economic advantages.

The remainder of the paper is structured as follows: the network model and message considerations are presented next. Subsequently, we develop a model for cost analysis, followed by a thorough investigation of the numerical findings and discussions. Finally, conclusions are drawn with future research.

## Network model and mobility messages

Here, we present a network model that provides an impression of the domain responsible for administrative purposes, including access networks and various entities within it. This model offers a general representation of the network structure and its components. Furthermore, we describe the messages used for mobility by IP mobility management protocols. These messages serve as the means of communication and coordination between network entities to manage and handle mobility-related operations effectively. By understanding the network model and mobility messages, we can gain insight into the functioning and behaviour of IP mobility management protocols within the administrative domain.

### Network model

**Fig 1:**
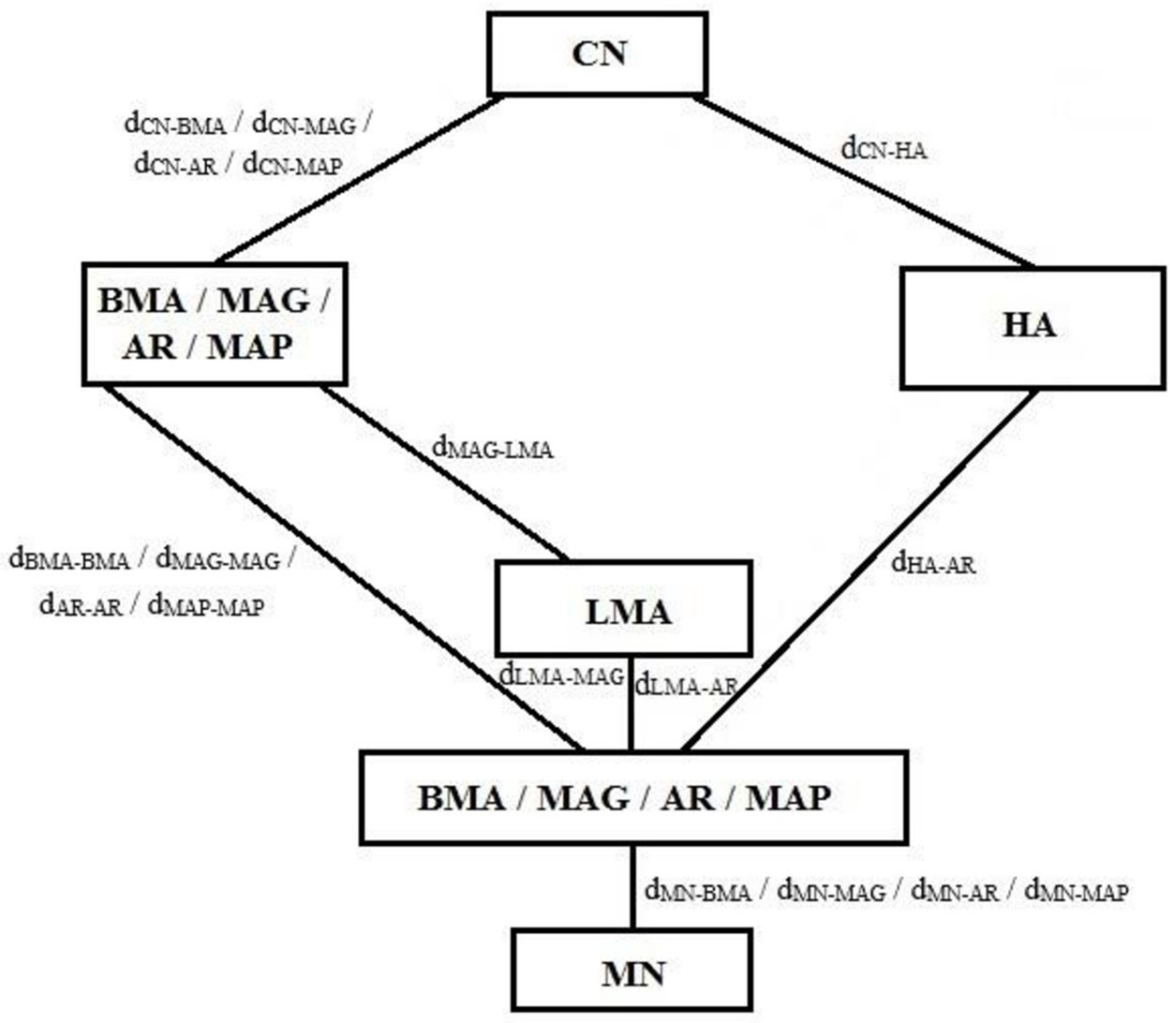
Network topology for performance evaluation. AR: access router, LMA: local mobility agent, MAG: mobile access gateway, MAP: mobility anchor point.

Fig 1 illustrates the network model employed for cost modelling, which corresponds to the model utilized in our previous work [1], as this research is an extension of the same study. The terms utilized in Fig 1, which illustrate the particular paths connecting the interacting entities, are explained in Table 2.

**Table 2:** Paths connecting entities.

Path Connection	Description
*dCN* − *BMA*/*dCN* − *MAG*/*dCN* − *AR*/*dCN* − *MAP*	The mean hop counts among *CN* and the *BMA*/*MAG*/*AR*/*MAP*
*dCN* − *HA*	The mean hop counts among *CN* and the *HA*
*dMAG* − *LMA*	The mean hop counts among *MAG* and the *LMA*
*dBMA* − *BMA*/*dMAG* − *MAG*/*dAR* − *AR*/*dMAP* − *MAP*/*dMAP* − *AR*	The mean hop counts among *BMA* and *BMA*, *MAG* and *MAG*, *AR* and *AR*, *MAP* and *MAP*, *MAP* and *AR*
*dLMA* − *MAG*	The mean hop counts among *LMA* and *MAG*
*dLMA* − *AR*	The mean hop counts among *LMA* and *AR*
*dHA* − *AR*	The mean hop counts among *HA* and *AR*
*dMN* − *BMA*/*dMN* − *MAG*/*dMN* − *AR*/*dMN* − *MAP*	The mean hop counts among mobile node (MN) and the *BMA*/*MAG*/*AR*/*MAP*

### Mobility messages

Messages required for MIPv6, PMIPv6, FMIPv6, HMIPv6 and the proposed TFMIPv6 approach with size expressed in bytes [28–30] and are given in Table 3.

**Table 3:** Messages and their sizes.

Messages	Description and Size
*MNBU* − *LMA*	The size of *NBU* (New Binding Update) message, forwarded to the *LMA* from the *MAG*, Size is 76
*MNBACK* − *LMA*	The size of *NBACK* (New Binding Acknowledgement) message size, Size is 76
*mLBU* − *MAP*	The size of Local Binding Update (LBU) message, forwarded to *MAP* from the *MN*, Size is 56
*mLBACK* − *MAP*	The size of Local Binding Acknowledgement (LBACK) message, forwarded from the *MAG* to the *LMA*, Size is 76
*mBU* − *CN*	The size of *BU* (Binding Update) message forwarded from the *MN* to the *CN*, Size is 66
*mBU* − *HA*	The size of the *BU* message forwarded from the *MN* to the *HA*, Size is 56
*mBAck* − *HA*	The size of the binding acknowledgement (*BAck*) message, Size is 56
*mHOTI*	The Home Test Init (*HoTI*) message, Size is 64
*mHOT*	The Home Test (*HoT*) message, Size is 74
*mCOTI*	The Care-of Test Init (*CoTI*) message, Size is 64
*mCOT*	The size of the Care-of Test (*CoT*) message, Size is 74

## Cost modeling for IP mobility management protocols

This section presents the construction of a cost model for evaluating and comparing IP mobility management protocols with the suggested TFMIPv6 framework. Our analysis focuses on a key cost factor, namely the signalling cost. We aim to comprehensively understand the implications and efficiency of different mobility management protocols. This enables us to make informed evaluations and comparisons between the protocols, facilitating decision-making regarding their implementation and utilization.

### Signaling cost

The signalling cost is the total signalling burden incurred during mobility-related operations. It encompasses the cumulative signalling burden associated with managing mobility in a network. If signalling cost is represented by $C_{BU}^{\left(.\right)}$, it can be found by multiplying the distance covered in each hop with the size of the mobility signalling message [28, 29].

#### Signaling cost for MIPv6

When using MIPv6, the *MN* sends *BU* messages to both the *CN* and the *HA* when its connection point changes. The signaling cost for MIPv6, denoted as MIPv6 $C_{BU}^{\left((MIPv6)\right)}$, consists of dual components: $C_{\left(BU-CN\right)}^{\left((MIPv6)\right)}$, which represents the signaling cost for *CN* registration, and $C_{\left(BU-HA\right)}^{\left((MIPv6)\right)}$, which represents the signaling cost for *HA* registration. The formula for $C_{BU}^{\left((MIPv6)\right)}$ can be expressed in Eq 1.

$$\begin{array}{c} C_{BU}^{\left(MIPv6\right)}=C_{BU-CN}^{\left(MIPv6\right)}+C_{BU-HA}^{\left(MIPv6\right)} \end{array}$$ (1)

where $C_{BU-HA}^{\left(MIPv6\right)}$ is expressed in Eq 2.

$$\begin{array}{c} C_{BU-HA}^{\left(MIPv6\right)}=m_{BU-HA}(\alpha d_{HA-AR}+\beta d_{AR-MN})+\\ m_{BAck-HA}(\alpha d_{HA-AR}+\beta d_{AR-MN}) \end{array}$$ (2)

The weighing factors *α* and *β* are employed to assess the stability of the link, with *α* representing the wired connection and *β* representing the wireless connection. These factors are used to emphasize the importance of link stability. Furthermore, the signalling cost for the registration of *CN*, denoted as $C_{\left(BU-CN\right)}^{\left((MIPv6)\right)}$, can be calculated using the following formula as shown in Eq 3.

$$\begin{array}{c} C_{BU-CN}^{\left(MIPv6\right)}=(m_{HoTI}+m_{HoT})(\alpha (d_{HA-AR}+d_{CN-HA})+\\ \beta d_{AR-MN})+(m_{CoTI}+m_{CoT})(\alpha d_{CN-AR}+\beta d_{AR-MN})+\\ m_{BU-CN}(\alpha d_{CN-AR}+\beta d_{AR-MN}) \end{array}$$ (3)

Nevertheless, the signalling cost mentioned earlier does not incorporate the overhead of signalling associated with *CN*′*s* BAck message, as MIPv6 specification does not require it [28].

#### Signaling cost for FMIPv6

FMIPv6 consists of dual modes: the predictive mode and the reactive mode. However, owing to handoff readiness required for *MN*, FMIPv6 incurs an additional cost of signalling [31]. Specifically, in the case of Predictive FMIPv6, there are three key aspects to consider: the signalling cost for handover preparation $C_{\left(BU-ready\right)}^{\left((Pre-FMIPv6)\right)}$, the signalling cost for *CN* registration $C_{\left(BU-CN\right)}^{\left((Pre-FMIPv6)\right)}$, and the signalling cost for *HA* registration $C_{\left(BU-HA\right)}^{\left((Pre-FMIPv6)\right)}$. If we denote the signaling cost [28, 32] of Pre-FMIPv6 as $C_{BU}^{\left((Pre-FMIPv6)\right)}$, it can be given in Eq 4.

$$\begin{array}{c} C_{BU}^{\left(Pre-FMIPv6\right)}=C_{BU-ready}^{\left(Pre-FMIPv6\right)}+C_{BU-CN}^{\left(Pre-FMIPv6\right)}+\\ C_{BU-HA}^{\left(Pre-FMIPv6\right)} \end{array}$$ (4)

Reactive FMIPv6 consists of three components: the signalling cost for handover preparation $C_{\left(BU-ready\right)}^{\left((Re-FMIPv6)\right)}$, the signalling cost for *CN* registration $C_{\left(BU-CN\right)}^{\left((Re-FMIPv6)\right)}$, and the signalling cost for *HA* registration $C_{\left(BU-HA\right)}^{\left((Re-FMIPv6)\right)}$. If we denote the signaling cost of Re-FMIPv6 as $C_{BU}^{\left((Re-FMIPv6)\right)}$, it can be given in Eq 5.

$$\begin{array}{c} C_{BU}^{\left(Re-FMIPv6\right)}=C_{BU-ready}^{\left(Re-FMIPv6\right)}+C_{BU-CN}^{\left(Re-FMIPv6\right)}+\\ C_{BU-HA}^{\left(Re-FMIPv6\right)} \end{array}$$ (5)

#### Signaling cost for HMIPv6

When a *MN* is in motion, it is managed locally by HMIPv6 [33] much like PMIPv6. After configuring a new access network, the MN solely needs to modify the *MAP* with its updated whereabouts since the *MAP* acts as the *MN*′*s* local *HA*. However, the mobility of the *MN* remains invisible to both the *HA* and the *CN*. In this case, the signaling cost of HMIPv6, denoted as $C_{BU}^{\left((HMIPv6)\right)}$ [20], can be expressed as can be given in Eq 6.

$$\begin{array}{c} C_{BU}^{\left(HMIPv6\right)}=m_{LBU-MAP}(\alpha d_{MAP-AR}+\beta d_{AR-MN})+\\ m_{LBAck-MAP}(\alpha d_{MAP-AR}+\beta d_{AR-MN)} \end{array}$$ (6)

Here, *m*_(*LBU*−*MAP*)_ represents the size of the local binding update (LBU) message transmitted from the MN to *MAP*. The parameters *α* and *β* represent the consideration criteria for wireless and wired links, respectively. *d*_(*MAP*−*AR*)_ denotes the mean number of hops between the *AR* and the *MAP*, while *d*_(*AR*−*MN*)_ signifies the mean number of hops between the *AR* and the *MN*. Additionally, *m*_(*LBAck*−*MAP*)_ refers to the size of the local binding acknowledgement message (*LBAck*) transmitted from the *MAP* to the *MN*.

#### Signaling cost for PMIPv6

Local management is carried out for a moving *MN* by PMIPv6 [34]. The *MN* utilizes mobility services entities provide to update its connection point within the designated area of PMIPv6. In the PMIPv6 area, no messages for signalling are transmitted by the *MN* specifically related to mobility. Consequently, the signaling cost of PMIPv6, denoted as $C_{BU}^{\left((PMIPv6)\right)}$ [28, 35] and can be given in Eq 7.

$$\begin{array}{c} C_{BU}^{\left(HMIPv6\right)}=2m_{PBU-LMA}\alpha d_{LMA-AR}+\\ 2m_{PBAck-LMA}\alpha d_{LMA-AR} \end{array}$$ (7)

Here, *m*_(*NBU*−*LMA*)_ represents the size of the *NBU* message sent from the *MAG* to the *LMA*. The parameter *α* is the consideration criteria for the wired link. *d*_(*LMA*−*AR*)_ signifies the mean number of hops between *LMA* and the *AR*. Additionally, *m*_(*NBack*−*LMA*)_ denotes the size of the NBack message transmitted from *LMA* to the *MAG*.

#### Signaling cost for TFMIPv6

For the suggested TFMIPv6 method, whenever the *MN* changes its place of attachment, it directs BU messages to both the BMA and the *CN*. The signaling cost of TFMIPv6, denoted as *C*_*B*_*U*^((*TFMIPv*6))^, consists of two components. The $C_{\left(BU-CN\right)}^{\left((FMIPv6)\right)}$ which is the cost involved in signaling for *CN*′*s* registration, and $C_{\left(BU-BMA\right)}^{\left((TFMIPv6)\right)}$ which is the cost involved in signaling for *BMA*′*s* registration. The $C_{BU}^{\left((TFMIPv6)\right)}$ can be calculated in Eq 8.

$$\begin{array}{c} C_{BU}^{\left(TFMIPv6\right)}=C_{BU-BMA}^{\left(TFMIPv6\right)}+C_{BU-CN}^{\left(TFMIPv6\right)} \end{array}$$ (8)

where, $C_{BU-BMA}^{\left(TFMIPv6\right)}$ and $C_{BU-CN}^{\left(TFMIPv6\right)}$ can be given in Eqs 9 and 10 respectively,

$$\begin{array}{c} C_{BU-BMA}^{\left(TFMIPv6\right)}=m_{PBU-BMA}(\alpha d_{BMA-BMA}+\\ \beta d_{BMA-CN})+m_{PBAck-BMA}(\alpha d_{BMA-BMA}\\ +\beta d_{BMA-CN}) \end{array}$$ (9)

and,

$$\begin{array}{c} C_{BU-CN}^{\left(TFMIPv6\right)}=(\alpha (d_{BMA-BMA}+d_{CN-BMA})+\\ \beta d_{BMA-MN})+(\alpha d_{CN-BMA}+\beta d_{BMA-MN})\\ +m_{BU-CN}(\alpha d_{CN-BMA}+\beta d_{BMA-MN}) \end{array}$$ (10)

### Packet delivery cost

The packet delivery cost is the overall extra data traffic that arises on routing paths because of packet delivery. We can represent the packet delivery cost as $C_{PD}^{\left((.)\right)}$, which is calculated by multiplying the hop distance with the size of the data packet [28].

#### Packet delivery cost for MIPv6

Data packets from the *CN* are sent directly to the *MN*′*s* current location [36] when using route optimization (*RO*). The packet delivery cost for MIPv6, denoted as $C_{PD}^{\left((MIPv6)\right)}$ [20, 28] is determined in Eq 11,

$$\begin{array}{c} C_{PD}^{\left(MIPv6\right)}=\omega \lambda_{s}E(S)P_{I}^{\left(MIPv6\right)} \end{array}$$ (11)

*ω* shows the data packets taking a longer route via the *HA* while the *CN*′*s* location update is ongoing. λ_*s*_ indicates how often new sessions are started by the *MN*, and *E*(*S*) is the average length of those sessions. $P_{I}^{\left((MIPv6)\right)}$ is the cost associated with using the indirect path option in MIPv6 can be given in Eq 12.

$$\begin{array}{c} P_{I}^{\left(MIPv6\right)}=\alpha d_{CN-HA}+\varpi \alpha d_{HA-AR}+\varpi \beta d_{AR-MN} \end{array}$$ (12)

Whereas *ϖ* represents the additional overhead caused by MIPv6 tunnelling. *d*_(*CN*−*HA*)_ shows the mean hop count among *CN* and *HA*, *d*_(*HA*−*AR*)_ shows the mean hop count among *HA* and the *AR*, *d*_(*AR*−*MN*)_ shows the mean hop count among *AR* and *MN*.

#### Packet delivery cost for FMIPv6

The *MN* receives data packets from the *CN* either directly or indirectly through different paths. In FMIPv6, to avoid data packet loss, a buffering mechanism is employed [31]. The cost of delivering packets for the predictive FMIPv6, denoted as $C_{PD}^{\left((PRE-FMIPv6)\right)}$ [20, 28, 32] is given in Eq 13.

$$\begin{array}{c} C_{PD}^{\left(PRE-FMIPv6\right)}=\omega \lambda_{s}E(S)P_{I}^{\left(PRE-FMIPv6\right)}+(1-\omega )\lambda_{s}\\ E(S)P_{D}^{\left(PRE-FMIPv6\right)} \end{array}$$ (13)

The $P_{D}^{\left((PRE-FMIPv6)\right)}$ represents the cost associated with a direct path in a predictive FMIPv6. When it comes to the packet delivery cost, the reactive FMIPv6 packet delivery cost $C_{PD}^{\left((RE-FMIPv6)\right)}$, is similar to that of predictive FMIPv6, $C_{PD}^{\left((PRE-FMIPv6)\right)}$. Thus, it can also be expressed in Eq 14 [28].

$$\begin{array}{c} C_{PD}^{\left(RE-FMIPv6\right)}=\omega \lambda_{s}E(S)P_{I}^{\left(RE-FMIPv6\right)}+(1-\omega )\lambda_{s}\\ E(S)P_{D}^{\left(RE-FMIPv6\right)} \end{array}$$ (14)

#### Packet delivery cost for HMIPv6

The *MN* receives packets from the *CN* while in the *MAP* domain. Usually, data packets are sent directly through the *HA* for the *MN*. However, the *MAP* tunnels the data packets for the *MN* [33] in certain cases. The cost of packet delivery of HMIPv6 $C_{PD}^{\left(HMIPv6\right)}$ is given as [20, 28]. The packet delivery cost of HMIPv6 can be computed using Eq 15.

$$\begin{array}{c} C_{PD}^{\left(HMIPv6\right)}=\lambda_{s}E(S)TP_{D}^{\left(HMIPv6\right)} \end{array}$$ (15)

where $PT_{D}^{\left((HMIPv6)\right)}$ represents the extra data transmission overhead incurred when tunnelling through the direct path in HMIPv6. This cost can be expressed in Eq 16.

$$\begin{array}{c} PT_{D}^{\left(HMIPv6\right)}=\varpi \alpha d_{MAP-AR}+\varpi \beta d_{AR-MN} \end{array}$$ (16)

where *d*_(*MAP*−*AR*)_ represents the average number of hops between the *MAP* and *AR*, and *d*_(*AR*−*MN*)_ indicates the average number of hops between the *AR* and the *MN*.

#### Packet delivery cost for PMIPv6

The *CN* sends data packets to the *MN*. The MAG receives these packets through tunnelling from the *LMA* [34]. The cost of packet delivery [34] of PMIPv6 $C_{PD}^{\left((PMIPv6)\right)}$ can be computed by using Eq 17.

$$\begin{array}{c} C_{PD}^{\left(PMIPv6\right)}=\lambda_{s}E(S)PT_{D}^{\left(PMIPv6\right)} \end{array}$$ (17)

Where $PT_{D}^{\left((PMIPv6)\right)}$ represents the additional data transmission overhead involved in direct path tunnelling of PMIPv6. This overhead can be given in Eq 18,

$$\begin{array}{c} PT_{D}^{\left(PMIPv6\right)}=\varpi \alpha d_{LMA-AR} \end{array}$$ (18)

where *d*_(*LMA*−*AR*)_ is the mean hop count among the *AR* and the *LMA*.

#### Packet delivery cost for TFMIPv6

The packets of data sent towards *MN* by *CN* are directed to the present position of the *MN* via *BMA*. The cost of packet delivery of TFMIPv6 $C_{PD}^{\left((TFMIPv6)\right)}$ can be given in Eq 19.

$$\begin{array}{c} C_{PD}^{\left(TFMIPv6\right)}=\lambda_{s}E(S)P^{\left(TFMIPv6\right)} \end{array}$$ (19)

Where λ_*s*_ represents the rate at which new sessions are initiated by the *MN*, *E*(*S*) denotes the average session length in terms of packets. *P*^((*TFMIPv*6))^ corresponds to the path cost and can be given in Eq 20.

$$\begin{array}{c} P^{\left(TFMIPv6\right)}=\alpha d_{CN-BMA}+\beta d_{BMA-MN} \end{array}$$ (20)

where *d*_(*CN*−*BMA*)_ represents the average number of hops between the *CN* and the *BMA*, and *d*_(*BMA*−*MN*)_ indicates the average hop count between the *BMA* and the *MN*. It’s important to note that the proposed approach eliminates the tunnelling overhead *ϖ*.

### Packet tunneling cost

Packet tunnelling cost *CPT*^(.)^ is quite similar to the packet delivery cost, but its primary purpose is to examine the extra load incurred during the tunnelling process. This cost is calculated by multiplying the distance covered in each hop with the size of the IPv6 tunnelling.

#### Packet tunneling cost for MIPv6

The cost of packet tunneling of MIPv6 *CPT*^(*MIPv*6)^ [28, 37] can be computed by using Eq 21.

$$\begin{array}{c} C_{PT}^{\left(MIPv6\right)}=\omega \lambda_{s}E(S)PT_{I}^{\left(MIPv6\right)}+\\ (1-\omega )\lambda_{s}E(S)PT_{D}^{\left(MIPv6\right)} \end{array}$$ (21)

where $PT_{I}^{\left(MIPv6\right)}$ represents the additional load caused by tunnelling through the indirect path and $PT_{D}^{\left(MIPv6\right)}$ represents the extra load incurred when tunnelling through the direct path and can be calculated in Eqs 22 and 23.

$$\begin{array}{c} PT_{I}^{\left(MIPv6\right)}=\varpi \alpha d_{HA-AR}+\varpi \beta d_{AR-MN} \end{array}$$ (22)

$$\begin{array}{c} PT_{D}^{\left(MIPv6\right)}=\varpi \alpha d_{CN-AR}+\varpi \beta d_{AR-MN} \end{array}$$ (23)

where *ϖ* represents the IPv6 overhead incurred during tunneling.

#### Packet tunneling cost for FMIPv6

The cost of packet tunneling of Predictive FMIPv6 [28]$C_{PT}^{\left(Pre-FMIPv6\right)}$ is expressed by using Eq 24.

$$\begin{array}{c} C_{PT}^{\left(Pre-FMIPv6\right)}=\omega \lambda_{s}E(S)PT_{I}^{\left(Pre-FMIPv6\right)}+\\ (1-\omega )\lambda_{s}E(S)PT_{D}^{\left(Pre-FMIPv6\right)} \end{array}$$ (24)

where $PT_{I}^{\left(Pre-FMIPv6\right)}$ stands for the additional load caused by tunnelling through the indirect path and $PT_{D}^{\left(Pre-FMIPv6\right)}$ represents the extra load incurred when tunnelling through the direct path [28]. These costs are determined by using Eqs 25 and 26.

$$\begin{array}{c} PT_{I}^{\left(Pre-FMIPv6\right)}=\alpha d_{CN-HA}+\varpi \alpha d_{HA-AR}+\\ 2\varpi \alpha d_{AR-AR}+\varpi \beta d_{AR-MN} \end{array}$$ (25)

$$\begin{array}{c} PT_{D}^{\left(Pre-FMIPv6\right)}=PT_{D}^{\left(MIPv6\right)} \end{array}$$ (26)

where $2\varpi \alpha d_{AR-AR}$ represents the extra load incurred due to tunnelling between the previous Access Router (*pAR*) and the new Access Router (*nAR*). Reactive FMIPv6 uses a buffering approach similar to predictive FMIPv6. When a *MN* undergoes a handoff procedure, the data packets intended for *MN* are buffered at the previous access router (*pAR*) and then sent through tunnelling to the new access router (*nAR*). As a result, the cost for packet tunnelling of Reactive FMIPv6, denoted as $C_{PT}^{\left(Re-FMIPv6\right)}$ is calculated in Eq 27.

$$\begin{array}{c} C_{PT}^{\left(Re-FMIPv6\right)}=C_{PT}^{\left(Pre-FMIPv6\right)} \end{array}$$ (27)

#### Packet tunneling cost for HMIPv6

The cost of packet tunneling [28, 38] of HMIPv6 $C_{PT}^{\left(HMIPv6\right)}$ and is determined by using Eq 28.

$$\begin{array}{c} C_{PT}^{\left(HMIPv6\right)}=\lambda_{s}E(S)PT_{D}^{\left(HMIPv6\right)} \end{array}$$ (28)

where $PT_{D}^{\left(HMIPv6\right)}$ represents the additional load incurred when tunnelling through the direct path in HMIPv6 and is given in Eq 29.

$$\begin{array}{c} PT_{D}^{\left(HMIPv6\right)}=\varpi \alpha d_{MAP-AR}+\varpi \beta d_{AR-MN} \end{array}$$ (29)

#### Packet tunneling cost for PMIPv6

The cost of tunnelling of packets of PMIPv6 $C_{PT}^{\left(PMIPv6\right)}$ is determined in Eq 30.

$$\begin{array}{c} C_{PT}^{\left(PMIPv6\right)}=\lambda_{s}E(S)PT_{D}^{\left(PMIPv6\right)} \end{array}$$ (30)

where $PT_{D}^{\left(PMIPv6\right)}$ represents the additional load incurred when tunnelling through the direct path in PMIPv6. Its value is specified in Eq 31.

$$\begin{array}{c} PT_{D}^{\left(PMIPv6\right)}=\varpi \alpha d_{LMA-AR} \end{array}$$ (31)

#### Packet tunneling cost for TFMIPv6

The cost for packet tunnelling of TFMIPv6 $C_{PT}^{\left(TFMIPv6\right)}$ is determined by the formula given in Eq 32.

$$\begin{array}{c} C_{PT}^{\left(TFMIPv6\right)}=\lambda_{s}E(S)PT^{\left(TFMIPv6\right)} \end{array}$$ (32)

where *PT*^(*TFMIPv*6)^ represents the additional load incurred when tunnelling through the suggested framework. Its value is determined in Eqs 33–35.

$$\begin{array}{c} PT^{\left(TFMIPv6\right)}=\varpi \alpha d_{CN-BMA}+\varpi \beta d_{BMA-MN} \end{array}$$ (33)

$$\begin{array}{c} PT^{\left(TFMIPv6\right)}=0.\alpha d_{CN-BMA}+0.\beta d_{BMA-MN} \end{array}$$ (34)

$$\begin{array}{c} PT^{\left(TFMIPv6\right)}=0 \end{array}$$ (35)

Since the proposed approach doesn’t involve any tunnelling as the data packets are sent directly to MN, the value of *PT*^(*TFMIPv*6)^ is calculated to be zero. As a result, the cost of tunnelling of TFMIPv6 is given in Eqs 36–38.

$$\begin{array}{c} C_{PT}^{\left(TFMIPv6\right)}=\lambda_{s}E(S)PT^{\left(TFMIPv6\right)} \end{array}$$ (36)

$$\begin{array}{c} C_{PT}^{\left(TFMIPv6\right)}=\lambda_{s}E(S).0 \end{array}$$ (37)

$$\begin{array}{c} C_{PT}^{\left(TFMIPv6\right)}=0 \end{array}$$ (38)

### Total cost

The total cost is represented as $C_{T}^{\left(.\right)}$, which can be expressed as the sum of two components: The Packet delivery cost $C_{PD}^{\left(.\right)}$ and signalling cost $C_{BU}^{\left(.\right)}$.

#### Total cost for MIPv6

The total cost of MIPv6 is obtained by adding the costs of signalling and packet delivery specific to MIPv6. This calculation is given in Eq 39.

$$\begin{array}{c} C_{T}^{\left(MIPv6\right)}=C_{BU}^{\left(MIPv6\right)}+C_{PD}^{\left(MIPv6\right)} \end{array}$$ (39)

#### Total cost for FMIPv6

The total cost for Pre-FMIPv6 and Re-FMIPv6 is determined by adding their individual costs of signalling and packet delivery. The specific calculations are provided in Eqs 40 and 41.

$$\begin{array}{c} C_{T}^{\left(Pre-FMIPv6\right)}=C_{BU}^{\left(Pre-FMIPv6\right)}+C_{PD}^{\left(PRe-FMIPv6\right)} \end{array}$$ (40)

$$\begin{array}{c} C_{T}^{\left(Re-FMIPv6\right)}=C_{BU}^{\left(Re-FMIPv6\right)}+C_{PD}^{\left(Re-FMIPv6\right)} \end{array}$$ (41)

#### Total cost for HMIPv6

The total cost for HMIPv6 is determined by adding its costs of signalling and packet delivery [28, 38]. The specific calculations can be computed by Eq 42.

$$\begin{array}{c} C_{T}^{\left(HMIPv6\right)}=C_{BU}^{\left(HMIPv6\right)}+C_{PD}^{\left(HMIPv6\right)} \end{array}$$ (42)

#### Total cost for PMIPv6

The total cost for PMIPv6 is determined by adding its individual costs of signalling and packet delivery [28, 35]. The specific calculations can be computed using Eq 43.

$$\begin{array}{c} C_{T}^{\left(PMIPv6\right)}=C_{BU}^{\left(PMIPv6\right)}+C_{PD}^{\left(PMIPv6\right)} \end{array}$$ (43)

#### Total cost for TFMIPv6

The total cost for TFMIPv6 is determined by adding its individual costs of signalling and packet delivery. The specific calculation is given by Eq 44.

$$\begin{array}{c} C_{T}^{\left(TFMIPv6\right)}=C_{BU}^{\left(TFMIPv6\right)}+C_{PD}^{\left(TFMIPv6\right)} \end{array}$$ (44)

## Results and discussion

This section shows the findings of the signalling cost analysis of the tunnel-free mobility management approach in contrast to the existing IETF standardized mobility management protocols. When a node changes its attachment point, it also experiences a shift in its logical address, and the connection to the previous link is considered lost. Mobility management protocols are designed to enable continuous active transmissions despite the modification in the node’s logical address. These protocols facilitate the mobility of nodes within a network, allowing them to move across various networks and access points concurrently sustaining uninterrupted communication sessions.

This study aims to examine the cost associated with existing mobility management protocols, specifically signalling costs. To accomplish this, a simulation environment was required to evaluate the performance of both established IETF approaches and the proposed approach regarding latency reduction, blocking probabilities, packet losses, and various cost factors. Network Simulator 2 (NS2) [36] was selected as the simulation platform for this research. NS2 was chosen due to its open-source nature, making it an accessible and widely used tool for conducting network simulations.

### Simulation parameters

In the simulation, different parameters are set up and adjusted to monitor how the network behaves and responds. Different parameters involved in the simulation process are given in Table 4, whereas for the sake of evaluation and comparison, the numerical analysis in this study utilizes system parameter values obtained from [28, 37–40]. Results are obtained in the context of costs involved in signalling the proposed framework.

**Table 4:** Simulation parameters and their values.

Simulation Parameters	Values
Propagation time of wired link	1.8ms
Simulation Area	1000 x 1000 m
Packet size	1kbytes
Buffer size in router	100kb
CBR source	2 for UDP/TCP
Simulation time	100sec
RS/ RA message size	52,80 bytes
BU/BA message size	56,56 bytes
NBU/NBAck message size	56,56 bytes
Mobility option for an address bound to the MN	20 bytes
PBU/ PBACK message size	76, 76 bytes
LBU/ LBACK message size	56, 76 bytes
Size of the Home Test Init (HoTI) message	64 bytes
Size of the Home Test (HoT) message	74 bytes
The size of the Care-of Test Init (CoTI) message	64 bytes
The size of the Care-of Test (CoT) message	74 bytes

### Signalling cost

**Fig 2:**
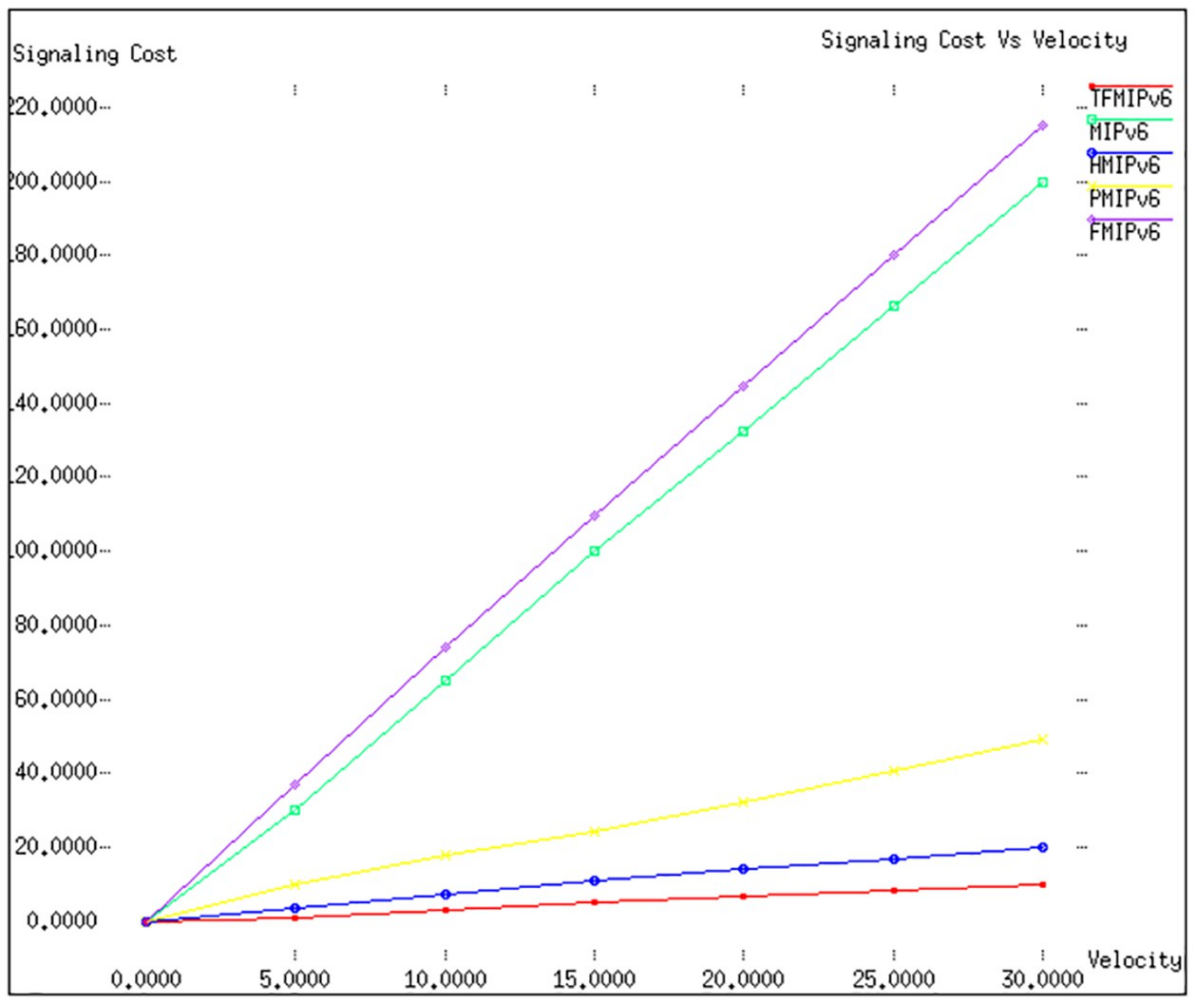
Signaling cost versus velocity.

**Fig 3:**
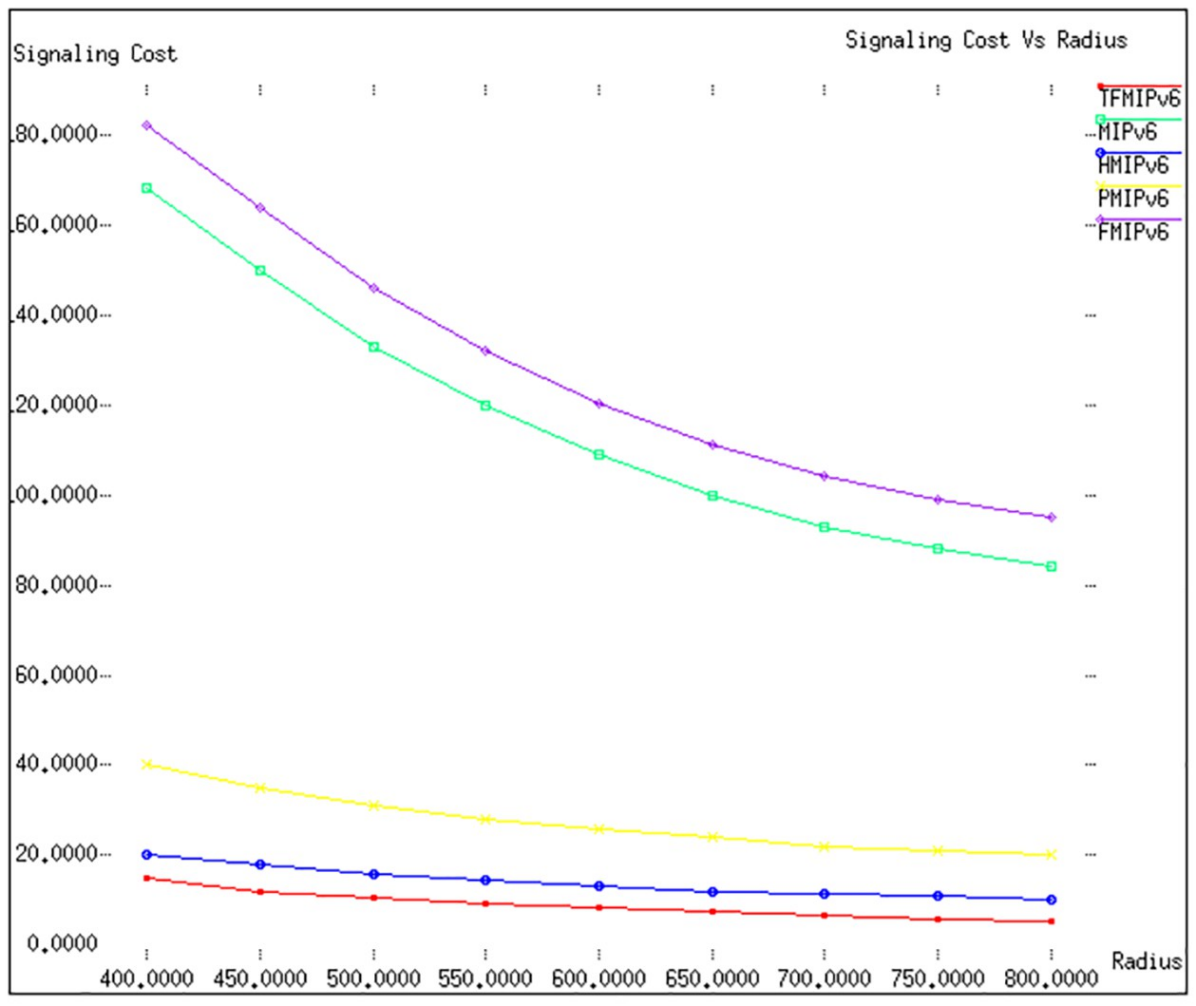
Signaling cost versus radius.

Figs 2 and 3 in our study offer a detailed comparison of the signalling costs associated with various mobility management protocols. This analysis is conducted under the parameters of a fixed radius (*R*) of 500 meters, with the velocities (v) of mobile nodes (*MN*) varying from 0 to 30 meters per second. In Fig 2, the effectiveness of the proposed *TFMIPv*6 protocol is highlighted. This protocol distinguishes itself by necessitating a significantly lower number of mobility signalling messages, which are crucial for supporting mobility services. As the velocity of the *MN* increases, an expected rise in signalling cost is observed across all protocols. However, the *TFMIPv*6 method consistently maintains lower signalling costs than its counterparts. Specifically, within a tunnel-free domain, the Binding Mobility Anchor (BMA) manages the *MN*′*s* mobility. During a transition in connection points by the *MN*, the exchange of messages is confined to the *BMA* and the *MN*, involving specific communications like Binding Acknowledgement (BAck), Binding Update (BU), New Binding Acknowledgement (NBACK), and New Binding Update (NBU).

In Fig 3, we delve into a scenario where the *MN*′*s* velocity is set at 20 meters per second, and the radius ranges between 400 and 800 meters. The results reinforce the superior performance of our suggested *TFMIPv*6 framework in comparison to other existing mobility management protocols. It’s important to note that in our simulation, *Re* − *FMIPv*6 was compared against other protocols instead of *Pre* − *FMIPv*6. This choice was informed by observations indicating *Re* − *FMIPv*6′*s* superior performance in certain contexts and comparable efficacy in others. In the protocols *MIPv*6 and *FMIPv*6, whenever the *MN* alters its connection point, it communicates this change by sending a Binding Update (BU) message to both the Correspondent Node (CN) and the Home Agent (HA). On the other hand, *HMIPv*6 demonstrates the second-best performance, followed by PMIPv6. The reason behind this ranking lies in the local management of the *MN* in both *HMIPv*6 and *PMIPv*6, which effectively reduces the signalling requirements for mobility. A surprising revelation from our analysis is the underperformance of *FMIPv*6, as depicted in both Figs 2 and 3. This is primarily attributed to the additional signalling demands imposed by its advanced mobility management, which facilitates buffering systems for seamless and rapid handovers. In conclusion, our detailed analysis and comparison of these protocols clearly demonstrate the efficiency and effectiveness of the *TFMIPv*6 method in managing mobility signalling, particularly in scenarios involving high velocities and varying radii. Summarizing the results from Figs 2 and 3 of our study into a tabular form. The Table 5 will capture the comparative analysis of signalling costs for various mobility management protocols under specified conditions.

**Table 5:** Comparative analysis of signaling costs for various mobility management protocols.

Protocol	Parameters	Velocity Range (0-30 m/s)	Observations
**TFMIPv6**	Radius: 500m	0-30 m/s	Consistently lower signaling costs across all velocities. Uses Binding Mobility Anchor (BMA) for managing mobility. Limited message exchange between BMA and MN.
**Re-FMIPv6**	Radius: 400-800m; Velocity: 20 m/s	0-30 m/s	Compared against other protocols. Shows superior performance in certain scenarios.
**MIPv6**	Radius: 500m	0-30 m/s	Binding Update (BU) message sent to both Correspondent Node (CN) and Home Agent (HA) when MN changes connection point.
**FMIPv6**	Radius: 500m	0-30 m/s	Higher signaling demands due to advanced mobility management for seamless handovers. Unexpected underperformance observed.
**HMIPv6**	Radius: 500m	0-30 m/s	Second-best performance. Local management of MN reduces signaling requirements.
**PMIPv6**	Radius: 500m	0-30 m/s	Similar to HMIPv6 in local management of MN, effectively reducing signaling needs.

Table 5 provides a concise comparison of the different protocols based on the results from the study, highlighting their performance in terms of signalling costs under varying velocities and radii. The *TFMIPv*6 protocol emerges as a more efficient option due to its lower signalling requirements, especially in high-velocity scenarios. To provide clarity on the mechanisms behind TFMIPv6’s reduced signaling costs, we have outlined the key features that distinguish TFMIPv6 from other protocols:

1. **Binding Mobility Anchor (BMA):** TFMIPv6 introduces the Binding Mobility Anchor (BMA), a central component responsible for managing mobility within the tunnel-free domain. The BMA efficiently exchanges only essential messages with the mobile node (MN), reducing the signaling burden by limiting the need to communicate with multiple network entities.
2. **Optimized Handover Procedures:** The Make-Before-Break (MBB) methodology ensures a smooth handover process by preparing the next access point before disconnecting from the current one. This proactive approach minimizes packet loss and avoids the need for additional signaling that reactive handover methods require.
3. **Tunnel-Free Domain:** TFMIPv6 eliminates tunneling by directly routing packets between the Correspondent Node (CN) and the MN through the BMA. This reduces signaling overhead compared to other protocols like MIPv6, where multiple messages are required for both Home Agent (HA) and CN registration.
4. **Efficient Message Exchange:** The signaling exchange in TFMIPv6 is limited to necessary messages, such as Binding Update (BU) and Binding Acknowledgement (BAck), which are directly handled between the BMA and MN. Other protocols like FMIPv6 require additional buffering or tunneling messages that increase signaling costs.
5. **Localized Management:** Unlike protocols that rely heavily on centralized entities, TFMIPv6 manages mobility locally through the BMA. This localized approach reduces signaling load by minimizing the distance and frequency of signaling message exchanges.
  These technical advantages of TFMIPv6 are clearly reflected in the reduced signaling costs demonstrated in our comparative analysis. By streamlining message exchanges, optimizing handover procedures, and eliminating tunneling, TFMIPv6 consistently delivers lower signaling costs than other mobility management protocols.

### Packet delivery cost

**Fig 4:**
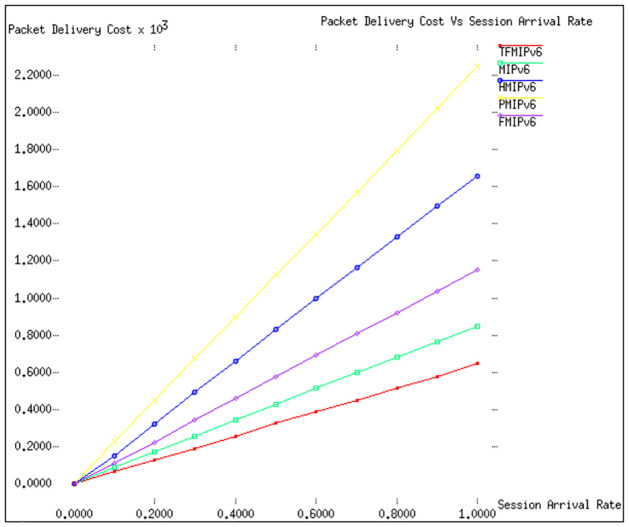
Packet delivery cost vs λ_*s*_.

**Fig 5:**
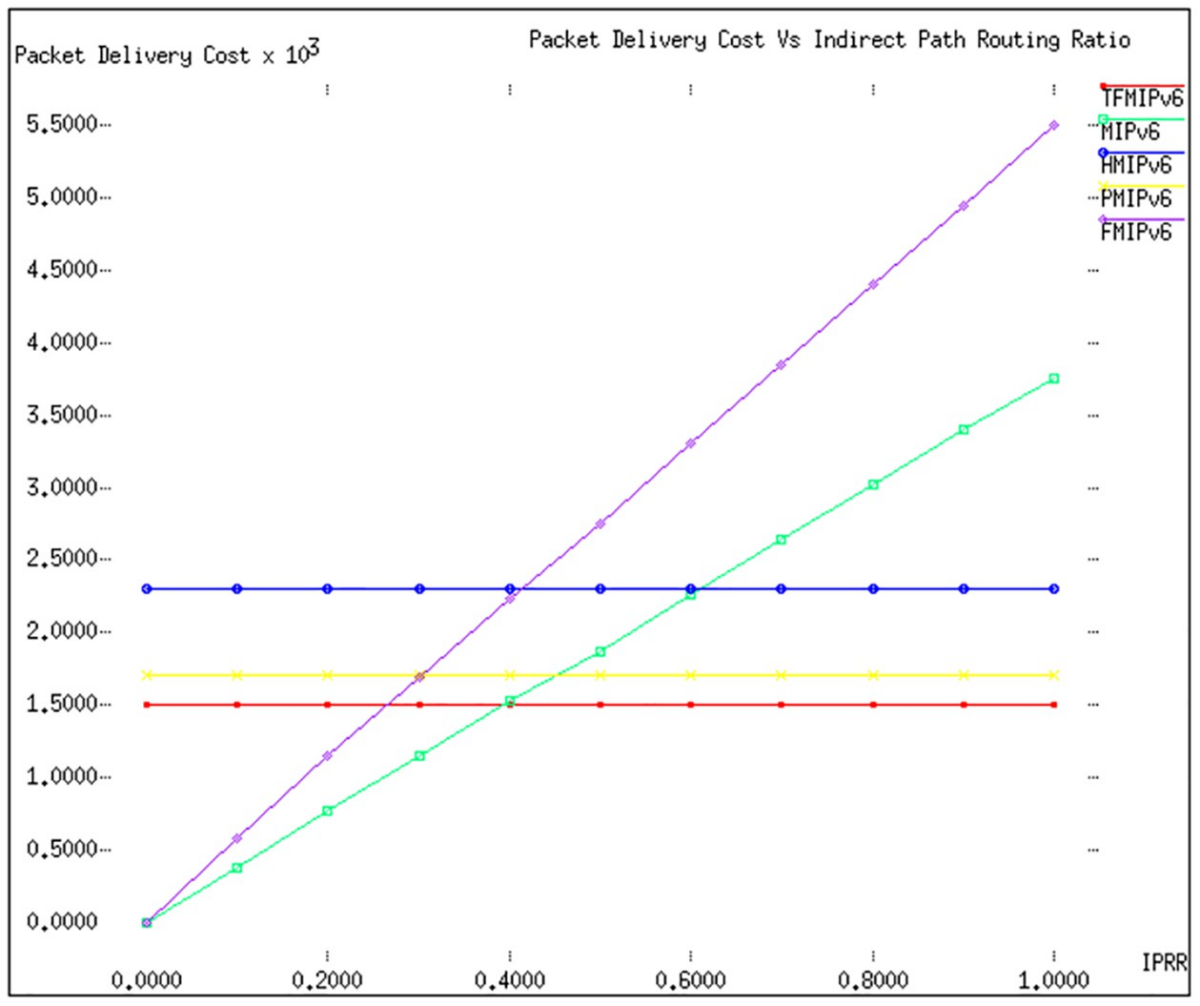
Packet delivery cost vs *ω*.

In our study, Figs 4 and 5 are pivotal in analyzing the cost implications associated with packet delivery across different mobility management protocols. These figures offer insights into how varying parameters impact the efficiency of these protocols in a real-world setting. Fig 4 focuses on the relationship between the packet delivery cost and different values of λ′*s* (arrival rate of packets), while keeping *ω* (the weight factor for secondary path usage) constant at 0.2 and *E*(*S*) (expected session duration) at 10. This graph clearly illustrates an upward trend in packet delivery costs as λ′*s* increases. Among the protocols evaluated, the TFMIPv6 method emerges as the most cost-effective, showcasing its ability to handle increasing packet arrival rates without significantly reducing delivery costs. *MIPv*6, while not as efficient as TFMIPv6, still performs commendably, securing a solid second place in terms of cost-effectiveness. Moving to Fig 5, the parameters are adjusted to set λ′*s* at 1 and *E*(*S*) at 10, with a variable *ω* ranging from 0.1 to 1. This configuration brings to light some intriguing observations. The *TFMIPv*6, *PMIPv*6, and *HMIPv*6 protocols show remarkable resilience to changes in *ω*, maintaining consistent packet delivery costs regardless of the variation in the weight factor. This suggests a robust design in these protocols, capable of adapting to different network conditions without incurring additional costs.

Conversely, *FMIPv*6 and *MIPv*6 exhibit a significant sensitivity to changes in *ω*. As *ω* increases, there’s a marked escalation in the packet delivery costs for these two protocols. This is primarily due to a higher proportion of data packets choosing the secondary route, which, in turn, increases the overall cost. Notably, *FMIPv*6 fares the worst in this scenario. This is attributed to the excessive tunnelling that *FMIPv*6 requires, which adds to the cost and complexity of packet delivery.

Overall, these findings from Figs 4 and 5 offer a comprehensive view of how different mobility management protocols perform under varying conditions. They underscore the cost-effectiveness and adaptability of the *TFMIPv*6 method, particularly in handling diverse network scenarios and traffic conditions. They also highlight areas where other protocols, such as *FMIPv*6 and *MIPv*6, may face challenges. This nuanced analysis provides valuable insights for network administrators and developers and guides future enhancements in mobility management protocols. Table 6 focuses on the packet delivery cost implications of various mobility management protocols under different conditions.

**Table 6:** Packet delivery cost implications of various mobility management protocols.

Figure	Parameter	Protocol	Observations and Performance
**4**	λ′*s* (Arrival Rate), *ω* = 0.2, *E*(*S*) = 10	**TFMIPv6**	Most cost-effective, handles increasing packet arrival rates well without a significant rise in delivery costs.
		**MIPv6**	Second in cost-effectiveness, performs well with increasing λ′*s*.
**5**	λ′*s* = 1, *E*(*S*) = 10, *ω* = 0.1 to 1	**TFMIPv6**	Shows resilience to changes in *ω*, maintaining consistent packet delivery costs.
		**PMIPv6**	Maintains consistent costs across varying *ω*, indicating robust design.
		**HMIPv6**	Similar performance to *TFMIPv*6 and *PMIPv*6, adaptable to *ω* changes without additional costs.
		**FMIPv6**	Sensitive to *ω* changes, leading to increased packet delivery costs, particularly affected by excessive tunnelling.
		**MIPv6**	Exhibits sensitivity to changes in *ω*, with costs escalating as *ω* increases.

Table 6 concisely summarizes the key findings from Figs 4 and 5, highlighting how different protocols perform in terms of packet delivery costs under various conditions. The *TFMIPv*6 protocol is cost-effective and adaptable, especially in scenarios with changing packet arrival rates and secondary path usage. Conversely, protocols like *FMIPv*6 and *MIPv*6 show sensitivity to these changes, resulting in higher packet delivery costs.

### Tunneling cost

**Fig 6:**
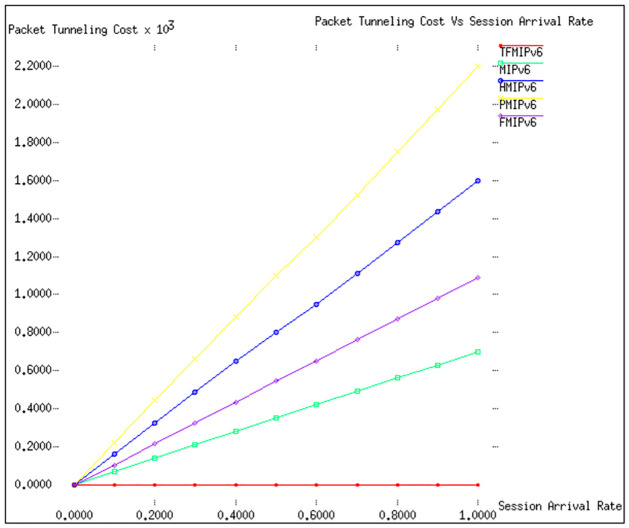
Packet tunneling cost vs λ_*s*_.

Fig 6 in our study presents a detailed and critical analysis of the tunnelling costs associated with various mobility management protocols, with a particular emphasis on how they compare to the proposed *TFMIPv*6 method. The settings for this analysis involve a constant *ω* (weight factor for secondary path usage) of 0.2 and *E*(*S*) (expected session duration) set at 10. While these parameters mirror those used in the packet delivery cost analysis, there’s a notable and significant difference in the context of packet tunnelling.

The key highlight from Fig 6 is the distinct advantage of the *TFMIPv*6 method in tunnelling costs. Unlike other protocols examined, *TFMIPv*6 is not affected by the costs associated with packet tunnelling. This is because *TFMIPv*6 employs a tunnel-free approach, effectively eliminating the additional overheads and complexities that tunnelling typically introduces.

In contrast, other protocols that rely on packet tunnelling for mobility management show varying degrees of cost increase due to this process. The tunnelling of packets, while a necessary component for these protocols, adds a layer of cost that cannot be overlooked. It involves additional steps in the data transmission process, potentially leading to increased latency and resource consumption. This distinction sets *TFMIPv*6 apart from its counterparts and underscores its efficiency and cost-effectiveness. By avoiding the tunnelling process, *TFMIPv*6 not only simplifies the mobility management process but also presents a more streamlined and economically viable option for handling mobile data transmission.

Overall, Fig 6 offers a compelling argument for the *TFMIPv*6 method, especially considering the cost implications of packet tunnelling in mobility management. This insight is particularly valuable for network designers and administrators seeking to optimize mobility management protocols while keeping operational costs in check. The tunnel-free nature of *TFMIPv*6 highlights its potential as a superior alternative in mobile network management.

Summarizing the findings from Fig 6 of the study into a tabular format focusing on the tunnelling costs associated with various mobility management protocols and their comparison with the *TFMIPv*6 method.

**Table 7:** Total cost analysis of various mobility management protocols based on the Session to Mobility Ratio (SMR).

Protocol	Parameter	Tunneling Costs	Remarks
**TFMIPv6**	(*ω* = 0.2, *E*(*S*) = 10)	None	Employing a tunnel-free approach, TFMIPv6 eliminates the costs and complexities typically associated with tunneling.
**Other Protocols**	(*ω* = 0.2, *E*(*S*) = 10)	Varies	Protocols relying on packet tunnelling show increased costs due to additional steps in data transmission and resource consumption.

Table 7 captures the essence of Fig 6, highlighting the significant advantage of the *TFMIPv*6 method in tunnelling costs. *TFMIPv*6 stands out due to its tunnel-free approach, which effectively removes the additional overheads and complexities inherent in other protocols that utilize packet tunnelling. This distinction emphasizes the efficiency and cost-effectiveness of *TFMIPv*6, making it an appealing option for network designers and administrators focused on optimizing mobility management protocols while managing operational costs.

### Total cost

**Fig 7:**
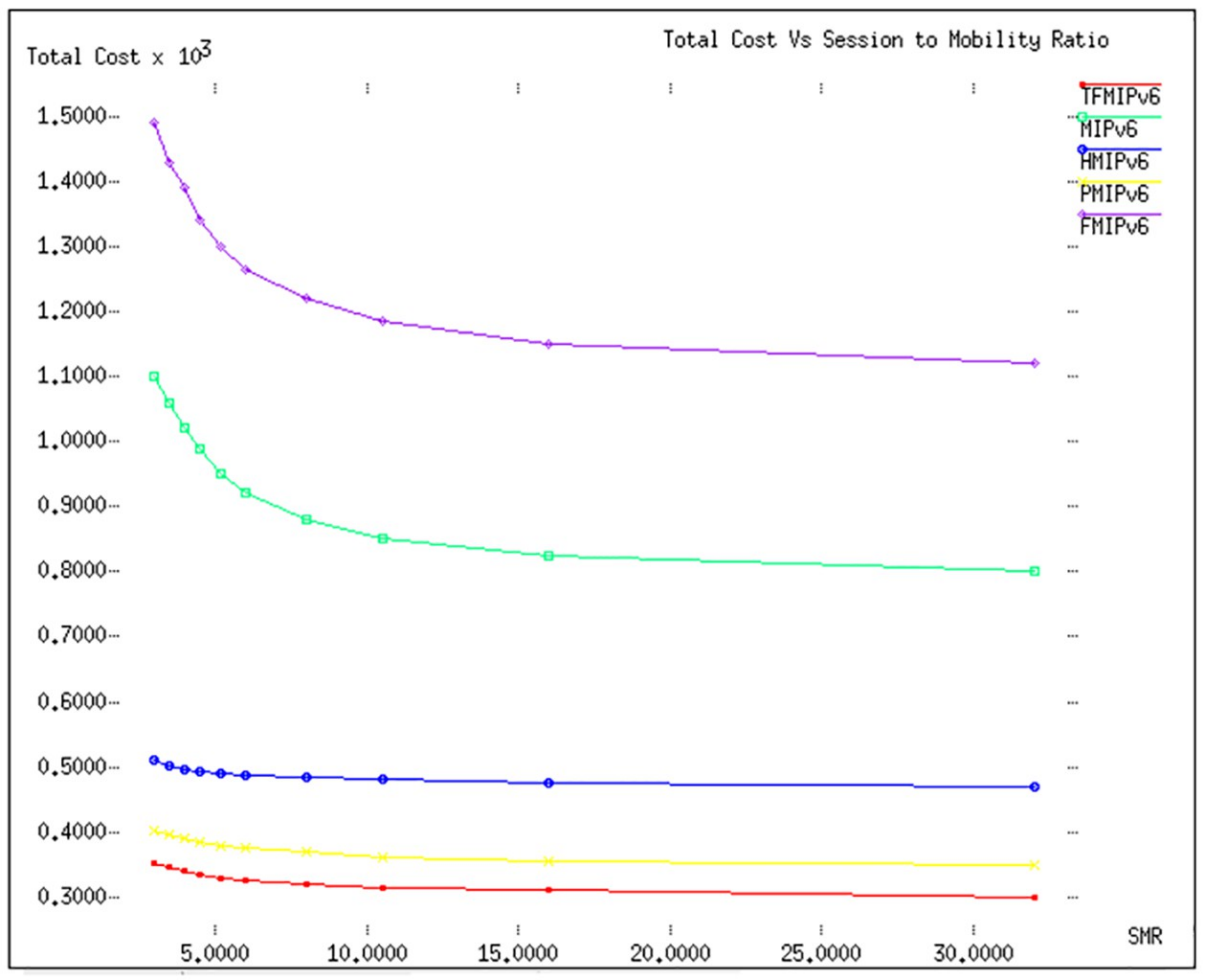
Total Cost Vs *S*_Φ_.

In our detailed analysis in Fig 7, we delve into the total cost associated with various mobility management protocols. The cornerstone of this analysis is the Session Mobility Ratio (SMR), denoted as *S*_Φ_. *SMR* is a critical metric, calculated from λ_*s*_/*μ*_*c*_, where λ_*s*_ represents the session arrival rate and *μ*_*c*_ denotes the mobility rate [29, 41, 42]. This metric, often employed in mobile networking performance evaluations, is akin to the call-to-mobility ratio, providing a balanced perspective on both the communication and mobility aspects of the network.

The configuration for this analysis in Fig 7 includes specific parameter settings: *ω* is fixed at 1, λ′*s* is set to 0.2, the radius (R) is maintained at 500 meters, and the velocity (v) varies from 5 to 50 meters per second. These parameters form the basis for comprehensively evaluating the total cost incurred by different protocols under various mobility conditions. The findings depicted in Fig 7 are particularly revealing. The *TFMIPv*6 strategy emerges as the most cost-effective approach among all the protocols evaluated. Its performance in managing costs associated with mobility management is notably superior, setting it apart from the alternatives. Following *TFMIPv*6, *PMIPv*6, and *HMIPv*6 also show commendable performance, indicating their effectiveness in managing costs, albeit not to the same extent as *TFMIPv*6. A key observation from this analysis is the resilience of the *TFMIPv*6 strategy against changes in *SMR*. Even as the *SMR* increases, which typically could impact the balance between session management and mobility management costs, the *TFMIPv*6 maintains its leading position. This consistency in performance, irrespective of the rise in *SMR*, underscores the robustness and efficiency of the *TFMIPv*6 strategy in managing total costs in mobile networks.

In summary, Fig 7 not only highlights the superior cost-efficiency of the *TFMIPv*6 strategy but also demonstrates its steady performance across varying network conditions. This insight is invaluable for network planners and engineers in designing and optimizing mobile networks for efficiency and cost-effectiveness, making *TFMIPv*6 a potentially preferred choice in mobility management solutions. Here’s a tabular summary of the results from Fig 7 of the study, focusing on the total cost analysis of various mobility management protocols based on the session-to-mobility ratio (SMR).

Table 8 encapsulates the findings from Fig 7, showcasing the *TFMIPv*6 strategy as the most cost-effective approach, particularly in managing mobility management costs under varying network conditions. *TFMIPv*6′*s* resilience against changes in *SMR* is a key highlight, indicating its robustness and efficiency in managing total costs in mobile networks. The table also compares the performance of PMIPv6 and *HMIPv*6, acknowledging their effectiveness but noting their limitations compared to *TFMIPv*6. The analysis provides essential insights for network planners and engineers in optimizing mobile networks for efficiency and cost-effectiveness. The strength and weakness of TFMIPv6 protocol are:

**Table 8:** Tunneling costs associated with various mobility management protocols and their comparison with the TFMIPv6 method.

Protocol	Parameter	Velocity Range (5-50 m/s)	Total Cost Analysis	Performance Against SMR Increase
**TFMIPv6**	(*ω* = 1, λ′*s* = 0.2, R = 500m)	5-50 m/s	Most cost-effective among all evaluated protocols.	Maintains cost-efficiency even with increased *SMR*, demonstrating resilience and robustness.
**PMIPv6**	(*ω* = 1, λ′*s* = 0.2, R = 500m)	5-50 m/s	Commendable performance, effectively managing costs.	Performs well, but not as efficiently as TFMIPv6.
**HMIPv6**	(*ω* = 1, λ′*s* = 0.2, R = 500m)	5-50 m/s	Good performance in managing costs.	Shows effectiveness in cost management but falls behind TFMIPv6.
**Other Protocols**	(*ω* = 1, λ′*s* = 0.2, R = 500m)	5-50 m/s	Higher total costs compared to TFMIPv6.	Less resilient against *SMR* increase, indicating higher cost implications.

### Strengths of TFMIPv6

- **Signaling Cost:** Our analysis of Figs 2 and 3 reveals that TFMIPv6 consistently maintains lower signaling costs than other protocols across varying velocities and radii. The efficiency stems from using a Base Mobility Agent (BMA) to handle mobile node mobility, minimizing unnecessary signaling exchanges.
- **Packet Delivery Cost:** According to Figs 4 and 5, TFMIPv6 demonstrates remarkable resilience in maintaining low packet delivery costs across different arrival rates and weight factors. Its adaptability to diverse network conditions further underscores its robust design.
- **Tunneling Cost:** As seen in Fig 6, TFMIPv6 eliminates tunneling costs by adopting a tunnel-free approach, reducing the overhead and complexity often associated with other protocols.
- **Total Cost:** Fig 7 highlights TFMIPv6’s cost-effectiveness across a range of Session to Mobility Ratios (SMR). It outperforms other protocols, demonstrating its resilience and robustness, particularly under varying network conditions.

### Limitations of TFMIPv6

- **Scalability Concerns:** The protocol’s performance might also be affected in extremely high-density mobile environments where signaling overhead may still accumulate despite reduced tunneling.
- **Complexity in Implementation:** Developing an efficient DMM protocol requires a precise design to balance signaling and data delivery costs. The implementation demands careful planning to ensure compatibility with existing network architectures.
- **Interoperability Issues:** Different vendors and standards may lead to interoperability challenges in hybrid environments where nodes use different protocols, requiring complex translation or adaptation layers.

### Comparison with other protocols

- **Re-FMIPv6:** It Shows superior performance in certain contexts but incurs higher signaling costs than TFMIPv6.
- **MIPv6 and FMIPv6:** Both protocols exhibit significant sensitivity to changes in weight factors, resulting in escalating packet delivery costs due to increased reliance on secondary paths.
- **HMIPv6 and PMIPv6:** Both demonstrate commendable performance in reducing signaling costs through local management of mobile nodes. However, their overall cost-effectiveness does not match TFMIPv6 due to persistent reliance on tunneling.

### Practical advantages

- TFMIPv6’s simplified signaling model makes it suitable for networks with fluctuating mobility demands.
- The tunnel-free approach is particularly advantageous for latency-sensitive applications, ensuring faster data packet delivery.
  These limitations highlight that while TFMIPv6 and similar DMM protocols have promising benefits, they require careful design and implementation to ensure robust, efficient, and secure mobility management.

## Conclusions and future research

In this paper, we introduce TFMIPv6, a novel tunnel-free protocol for distributed mobility management. Through extensive comparative analysis and simulation, our results demonstrate that TFMIPv6 significantly reduces signaling, packet delivery, tunneling, and total costs compared to other existing protocols. The key findings from our study include: (1). Signaling Costs: TFMIPv6 achieves up to 50% reduction in signaling costs due to its use of the Binding Mobility Anchor (BMA), which confines message exchanges to a localized domain. The Make-Before-Break (MBB) methodology also ensures efficient handovers, reducing signaling overhead during network transitions. (2). Packet Delivery Costs: By directly routing packets through the BMA and eliminating tunneling, TFMIPv6 minimizes delivery delays and achieves a 23% reduction in packet delivery costs. (3). Tunneling Costs: TFMIPv6 eliminates tunneling entirely, reducing the complexity and cost often associated with other protocols. (4). Total Costs: With improvements in signaling, packet delivery, and tunneling, TFMIPv6 achieves a 13% reduction in total costs, proving its cost-effectiveness and robustness.

### Academic implications

The TFMIPv6 protocol presents a practical and scalable solution for distributed mobility management in IPv6 networks. Its architecture serves as a valuable framework for designing future protocols, emphasizing localized management and efficient handover procedures. Significant reductions in signaling, packet delivery, and tunneling costs highlight their potential for practical applications in real-world networks.

### Future work

Although TFMIPv6 provides substantial improvements, further research is required to address some limitations and enhance the protocol’s scalability and security. Future directions include:

1. **Handover Optimization:** Investigating advanced handover techniques to optimize the process in extremely high-density networks and frequently mobile nodes.
2. **Load Balancing:** Developing scalable and distributed methods to balance the load across multiple BMAs, ensuring seamless operation in highly mobile environments.
3. **Security Enhancements:** Implementing additional security mechanisms to protect against vulnerabilities that could arise from localized management.
4. **Network Topology Impact:** Analyzing the impact of varying network topologies and node densities on TFMIPv6 to ensure robustness and adaptability.

These future research directions will comprehensively explore TFMIPv6’s potential and help refine it into a versatile, next-generation solution for distributed mobility management.
